# Prognostic Value and Risk Factors of Treatment-Related Lymphopenia in Malignant Glioma Patients Treated With Chemoradiotherapy: A Systematic Review and Meta-Analysis

**DOI:** 10.3389/fneur.2021.726561

**Published:** 2022-01-04

**Authors:** Yongchao Zhang, Shichao Chen, Hualei Chen, Shanshan Chen, Zhen Li, Enshan Feng, Wei Li

**Affiliations:** ^1^Cancer Center, Beijing Ditan Hospital, Capital Medical University, Beijing, China; ^2^Neurosurgery Department, Beijing Ditan Hospital, Capital Medical University, Beijing, China; ^3^Emergency Department, Beijing Youan Hospital, Capital Medical University, Beijing, China

**Keywords:** glioma, lymphopenia, chemoradiation, meta-analysis, temozolomide

## Abstract

**Background:** Immunotherapy has shown promising therapeutic efficacy in various cancers but not gliomas. Circulating lymphocytes play critical roles in cancer control and responses to immune checkpoint inhibitors. Treatment-related lymphopenia has been associated with poor survival in patients with various tumors. This meta-analysis evaluated the risk and impact of lymphopenia in patients with glioma.

**Methods:** The PubMed, Embase, Web of Science, and Cochrane Library databases were comprehensively searched. Eligible studies were included if they reported the incidence and risk factors of lymphopenia and the impact of lymphopenia on survival. Stata 16.0 was used for this meta-analysis.

**Results:** A total of 21 studies were included in the final systematic review and 20 were included in the quantitative analysis. The overall incidence of grade III/IV lymphopenia was 31.6% [95% confidence interval (CI), 22.3–40.8%]. Pooled results based on pathology of glioma revealed that the incidence in astrocytoma and astrocytoma oligodendroglioma patients was 20.2% (95% CI:5.9–34.4%), and the incidence in glioblastoma patients was 27.6% (95% CI:16.2–38.9%). Lymphopenia was associated with poor overall survival (hazard ratio, 1.99; 95% CI, 1.74–2.27; *P*< *0.001*) compared to no lymphopenia. Brain receiving radiation dose of 20 or 25 Gy, female sex, older age, lower baseline lymphocyte count, and dexamethasone dose > 2 mg instead of baseline use were risk factors for lymphopenia.

**Conclusions:** Treatment-related lymphopenia was associated with decreased survival in patients with glioma. Optimization of chemoradiation regimens, particularly in patients with concurrent risk factors, can reduce lymphopenia and potentially improve survival in the era of immunotherapy.

## Introduction

Gliomas are the most common type of central nervous system tumors among adults (1). Although glioma patients receive a combination of radiation therapy (RT) and chemotherapy as part of their treatment paradigm, either concomitantly and/or sequentially, the overall survival (OS) in cases of high-grade glioma, such as glioblastoma (GBM) remains poor (2). In recent years, immune checkpoint inhibitors have been effective for the treatment of various tumors, especially melanoma and non-small cell lung cancer (3, 4). However, immunotherapy presents a major challenge in glioma treatment because the brain is an immune-privileged organ. Increased understanding of immune characteristics during and after glioma treatment may provide insight into the optimal application of immunotherapeutic modalities.

Circulating lymphocytes have long been considered primary effector cells in the anti-tumor response, and a lack of lymphocytes could weaken the immune system's ability to eliminate tumor cells (5, 6). RT, temozolomide (TMZ), and glucocorticoids are routinely used to treat patients with high-grade gliomas. Each treatment causes lymphocyte toxicity. However, the concomitant use of these treatments is inevitable in clinical practice, thereby further inducing substantial lymphopenia, intensive immunosuppression, and opportunistic infections.

Lymphocytes are particularly sensitive to RT. Previous studies have demonstrated that lethal doses that decrease the surviving fraction by 50 and 90% are 2 and 3 Gy, respectively (7). A mathematical model revealed that the mean dose to circulating lymphocytes was 2.2 Gy, and 99% of lymphocytes received ≥ 0.5 Gy after a routine course of 30 fractions of 2 Gy RT (8). An observational study reported that a reduction of CD4 counts to <200/mm^3^ was observed in 17 (24%) of 70 patients following RT and glucocorticoids before the TMZ era (9). Indeed, recent studies and meta-analyses have reported that RT-induced lymphopenia is associated with a decreased survival in various cancers, including lung cancer, esophageal squamous cell carcinoma, and pancreatic cancer (10, 11). Moreover, severe and persistent lymphopenia has also been observed in patients with high-grade glioma after RT and TMZ (12, 13). Ongoing clinical trials are aiming to add nivolumab, an immune checkpoint inhibitor against programmed cell death 1 (PD-1), a pathway that downregulates the immune system, to RT [Checkmate 498, (NCT02617589)] or RT/TMZ [Checkmate 548 (NCT02667587)]. However, the latest results have suggested that nivolumab + RT or nivolumab + RT + TMZ did not show better efficacy than standard treatment, possibly due to lymphopenia (14).

A few recent studies have reported a relationship between iatrogenic lymphopenia and clinical outcomes in glioma patients (15, 16). Given the variability in lymphocyte counts among the general population and the small sample sizes, we performed this meta-analysis to list and assess studies of treatment-related lymphopenia in patients with newly diagnosed glioma and identify dosimetric and other risk factors for lymphopenia.

## Materials and Methods

### Search Strategies

The meta-analysis followed the guidelines of PRISMA (Preferred Reporting Items for Systematic Reviews and Meta-Analyses). A comprehensive electronic search was conducted using PubMed, EMBASE, Cochrane Library, and the Web of Science up to April 18, 2021. The search strategy was based on the following key words: “glioma,” “radiation,” “temozolomide,” “lymphopenia.” Details of the retrieval strategy are provided in Supplementary Table 1. The references in the identified studies were also traced to other relevant studies.

### Study Selection Criteria

The inclusion criteria for the study were as follows:

(1) Patients newly diagnosed with glioma
(2) Patients treated with concomitant chemoradiotherapy with TMZ followed by adjuvant TMZ
(3) Having data on treatment-related lymphopenia
(4) Sufficient data were provided to calculate hazard ratios (HRs) and 95% confidence intervals (CIs)
(5) Prospective or retrospective studies
(6) Articles were published in full texts, excluding the following:
  a. Case reports, animal experiments, conference abstracts, editorials, and reviews
  b. Studies with insufficient information to evaluate HRs and 95% CIs
  c. Patients who were included in identified articles in immunodeficiency states or using immune checkpoint inhibitors.
  d. Studies that were not communicated in English

### Data Extraction and Quality Assessment

Two investigators independently screened the studies that met our inclusion criteria and extracted the relevant information. Disagreements were resolved through discussions with an independent expert. The following information was extracted: first author's name, publication year, study design, country, sample size, age, sex, pathological type, treatment regimen, the cut-off to categorize high and low lymphocyte level, steroid use, HRs for OS, and 95% CIs. The incidence of severe lymphopenia was characterized based on and grade 3 or higher as reported by each studies using National Cancer Institute's Common Terminology Criteria for Adverse Events (CTCAE) definitions. The Quality Assessment of the Newcastle-Ottawa Scale (NOS) was used to evaluate the quality of eligible studies.

### Statistical Analysis

For each study, the proportion of patients with severe lymphopenia was calculated, and a 95% exact confidence interval was derived. HRs with their 95% CIs from the included studies were used to calculate the pooled HR. Heterogeneity was evaluated using the Higgins I^2^ statistic, and *I*^2^ > 50% was defined as significant heterogeneity. A fixed effect model or random effect model was used according to the heterogeneity of the pooled results. The data were synthesized using a fixed-effects model with *I*^2^ <50%. Otherwise, a random-effect model was used. All statistical tests were two-sided, and statistical significance was defined as *p* < 0.05. Pooled data were analyzed using STATA 16.0 (Stata Corp, College Station, TX).

## Results

### Study Selection and Characteristics

**Figure 1:**
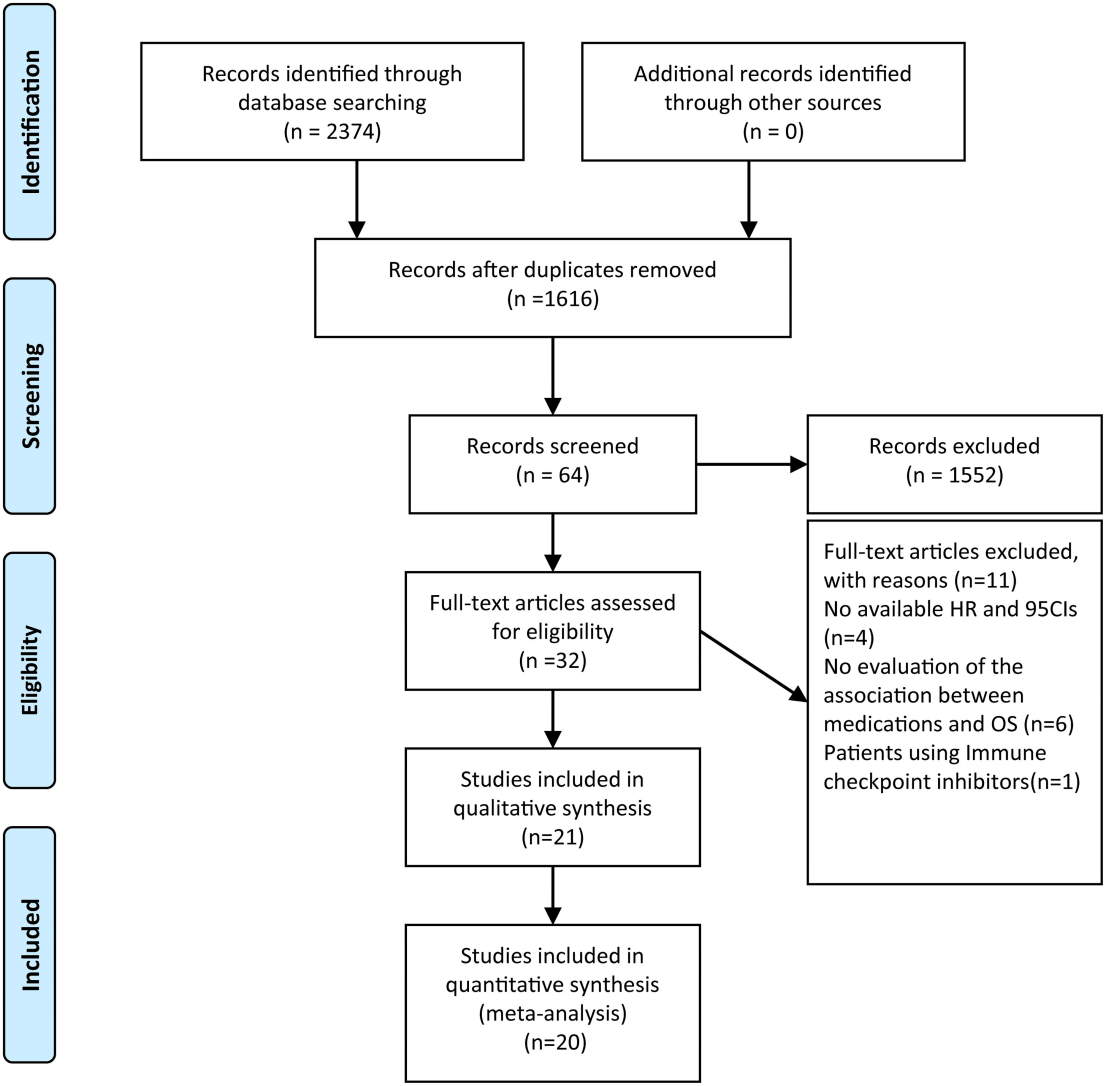
Flow chart of literature search and study selection. After carefully reviewed 21 studies were included in the final systematic review and 20 in the quantitative analysis.

A flow diagram of the literature selection process is shown in Figure 1. Our search yielded 2,374 relevant hits from the selected databases. A total of 1,616 records were included after duplicate removal. Among them, 1,552 were excluded after the titles and abstracts screening, leaving 64 relevant articles. The full-text articles of these 64 studies were reviewed; of them, 21 met the inclusion criteria. A total of 15 studies (12, 17–30) that evaluated the incidence of severe lymphopenia and six (12, 13, 15, 16, 31, 32) that evaluated the prognostic impact of lymphopenia on OS were selected. Furthermore, five studies (21, 30–33) that reported risk factors for lymphopenia on multivariate analysis were also included. As Mohan et al. (30) reported the incidence of lymphopenia in two cohorts in which patients were treated with proton therapy or X-ray therapy, we termed them “Mohan 2021 cohort 1” and “Mohan 2021 cohort 2,” respectively. The characteristics of the included studies are summarized in Tables 1, 2.

**Table 1:** The main characteristics of studies included in this meta-analysis for incidence.

**References**	**Country**	**Study design**	**Pathology**	**Age**	**Treatment**	**Severe lymphopenia**	**Sample size**	**NOS**
Stupp et al. (17)	Switzerland	Prospective	GBM	52 (24–70)	TMZ (75 mg/m2/d * 7 d/wk for 6 weeks) concomitant with fractionated RT (60 Gy total dose: 2 Gy * 5 d/wk for 6 weeks) + temozolomide (200 mg/m2/d *5 days, every 28 days for six cycles).	49 (80%)	62	8
De Sanctis et al. (18)	Italy	Prospective	GBM	61 (31–78)	RT (60Gy, 2Gy/day) and concomitant treatment with TMZ (75 mg/m2/day) and adjuvant TMZ (200 mg/m2/day for 5 days/28 days)	13 (30%)	43	7
Brandes et al. (19)	Italy	Prospective	GBM	68 (65–82)	RT (60 Gy in 30 fractions over 6 weeks) plus TMZ (75 mg/m2/day), followed by 12 maintenance TMZ cycles (150 mg/m2 once a day for 5 consecutive days every 28 days)	1 (2%)	58	7
Clarke et al. (20)	US	Prospective	GBM	56.3 (21–71)	Standard radiotherapy with concurrent daily TMZ followed by six adjuvant cycles of either dose-dense (150 mg/m2 days 1 to 7 and 15 to 21) or metronomic (50 mg/m2 continuous daily) TMZ.	20 (23.5%)	85	7
Ishikawa et al. (21)	Japan	Retrospective	AA GBM	36–75 years (mean 58.6 years)	RT 60 Gy in 2-Gy fractions on 5 days a week for 6 weeks in 20 patients, 45 Gy in 3-Gy fractions in 3 patients, total doses of 61.2 and 50 Gy in 1 patient each, and a total dose of 97 Gy with a proton beam in 3 patients. Concomitant TMZ at a daily dose of 75 mg/m2 for 7 days	23 (68%)	28	5
Grossman et al. (12)	US	Prospective	AA AOA GBM	57 (28–85)	Standard radiation and TMZ treatment.	36 (37%)	96	7
Minniti et al. (22)	Italy	Retrospective	GBM	73.2 (70–80)	Patients received focal RT plus concomitant daily TMZ, followed by adjuvant TMZ.	16 (19%)	83	6
Cohen et al. (23)	US	Prospective	AA GBM	at least 3 and <22	Concomitant chemoradiotherapy with TMZ followed by adjuvant chemotherapy with TMZ.	32 (36%)	90	7
Minniti et al. (24)	Italy	Retrospective	AA	43 (20–71)	RT 59.4–60 Gy delivered in 30–33 fractions of 1.8–2 Gy over a period of 6–6 1/2 weeks, started within 4–6 weeks from surgery. Adjuvant TMZ 150–200 mg/m2 for 5 days every 28 days up to 6–12 cycles.	16(17 %)	97	6
Saito et al. (25)	Japan	Retrospective	GBM	≥65 (*n* = 27) <65 (*n* = 49)	Standard treatment	33 (43.4%)	76	6
Nayak et al. (26)	US	Retrospective	AA AOA	48 years (range 28–74)	Concurrent RT (60 Gy over 6 weeks) and TMZ (75 mg/m2), and six adjuvant either dose-dense (150 mg/m2, days 1–7, 15–21) or metronomic (50 mg/m2, days 1–28) TMZ.	15 (75%)	20	5
Kim et al. (27)	Koraa	Retrospective	GBM	57.5 years (range, 20–86 years).	167 patients (76.3%) CCRT/TMZ-TMZ (60 Gy of radiotherapy in 30 fractions and 6 cycles of adjuvant TMZ); 52 patients (23.7%) were treated with hypo-CCRT/TMZ-TMZ (45 Gy of radiotherapy in 15 fractions and 6 cycles of adjuvant TMZ).	41 (5.5%)	750	5
Kim et al. (27), Perry et al. (28)	Multiple	Perspective	GBM	65–70 83 71–75 117 ≥76 81	Radiation for a total dose of 40.05 Gy, administered in 15 daily fractions over a period of 3 weeks	73 (27.2%)	281	6
Lin et al. (29)	Us	Retrospect	Astrocytoma Oligodendroglioma	42.8 (20.5–73.3)	IMRT or 3DCRT. Grade II gliomas were typically treated to 45–54 Gy, and grade 3 RT 59.4–63 Gy	9 (10%)	91	5
Mohan et al. Group 1 (30)	US	Retrospective	GBM	55.1 (10.7)	Proton therapy with TMZ	4 (14%)	28	7
Mohan et al. Group 2 (30)	US	Retrospective	GBM	51.3 (13)	X-ray (photon) therapy with TMZ	22 (39%)	56	7

*GBM, Glioblastoma; AA, anaplastic astrocytoma; AOA, anaplastic oligo-astrocytoma; TLC, total lymphocyte count; SFRT, standard-field radiation therapy; LFRT, limited-field radiation therapy; TMZ, temozolomide; CCRT, concomitant chemoradiotherapy; IMRT, intensity-modulated radiation therapy; 3DRT, three-dimensional conformal radiation therapy; NOS, Newcastle-Ottawa Scale*.

**Table 2:** The main characteristics of studies included in this meta-analysis for survival.

**ID**	**Country**	**Study design**	**Pathology**	**Age**	**Sex (male/ female)**	**Treatment**	**Baseline TLC, media*n* (range)**	**Sample size**	**Definition of lymphopenia**	**Steroids use *n* (%)**	**NOS**
Grossman et al. (12)	US	Prospective	anaplastic astrocytoma anaplastic oligodendroglioma GBM	57 (28–85)	48/48	Standard radiation and temozolomide treatment.	1,418 (331–4,736)	96	TLC <500 cells/mm3 at 2 month	79 (82%)	7
Mendez et al. (13)	Us	Retrospective	GBM	71 (65–86)	34/38	31% of patients RT <45 Gy and 90 % of patients received TMZ.	1,100 (300–3,200)	72	TLC <500 cells/mm3 at 2 month	61 (84%)	6
Rudra et al. (31)	Us	Retrospective	GBM	57 (21–82)	127/83	164 SFRT with TMZ 46 LFRT with TMZ	1,400 (300–4,200)	210	TLC <500 cells/mm3 within 3 month	>4 mg/day 42 (20%)	6
Kim et al. (15)	Korea	Retrospective	GBM	54.2 (16.0–83.0)	133/86	167 CCRT/TMZ-TMZ, 52 hypo-CCRT/TMZ-TMZ	1,780 (403–5,489)	219	TLC <500 cells/mm3 within 3 month	153 (69.8%)	6
Ahn et al. (16)	Korea	Retrospective	GBM	59.0 (50.0–66.0)	48/49	concomitant chemoradiation	1,578(1,237–2,101)	97	TLC <1,200 cells/mm3	25 (25.8%)	5
Lee et al. (32)	Korea	Retrospective	GBM	59.0 (20–79)	67/58	3DRT or IMRT with concomitant TMZ	1,583 (256–4,950)	125	TLC <1,000 cells/μL	36 (28.8%)	5

*GBM, Glioblastoma; TLC, total lymphocyte count; SFRT, standard-field radiation therapy; LFRT, limited-field radiation therapy; TMZ, temozolomide; CCRT, concomitant chemoradiotherapy; IMRT, intensity-modulated radiation therapy; 3DRT, three-dimensional conformal radiation therapy; NOS, Newcastle-Ottawa Scale*.

### Incidence of Severe Lymphopenia

**Figure 2:**
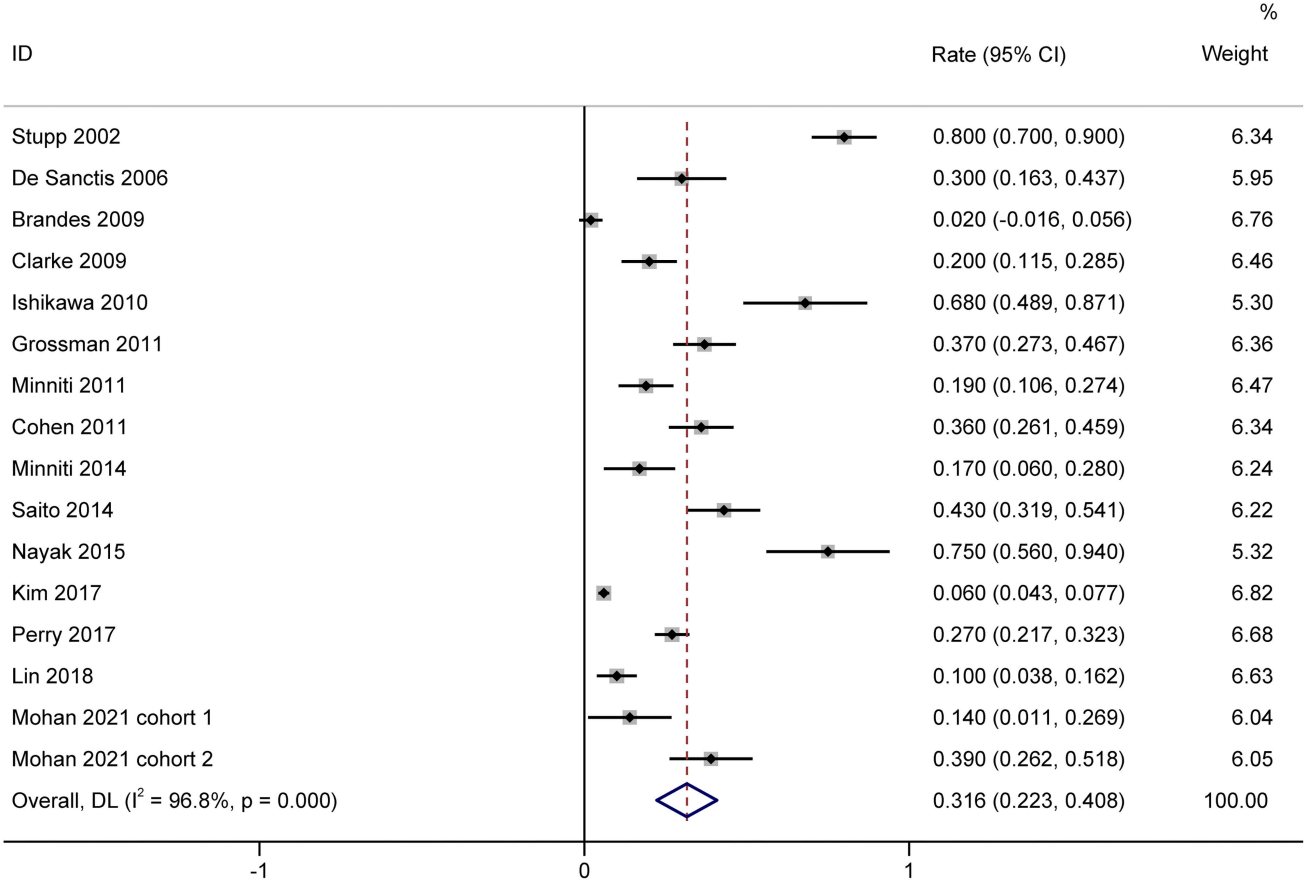
Forest plots of the incidence rate of severe lymphopenia and 95% CIs in glioma patients. Weights are from random-effects model.

**Figure 3:**
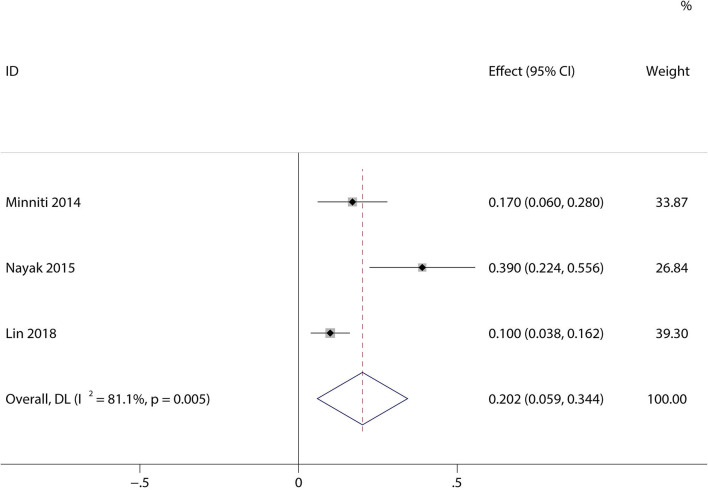
Forest plots of the incidence rate of severe lymphopenia and 95% CIs in astrocytoma and astrocytoma oligodendroglioma patients. Weights are from random-effects model.

**Figure 4:**
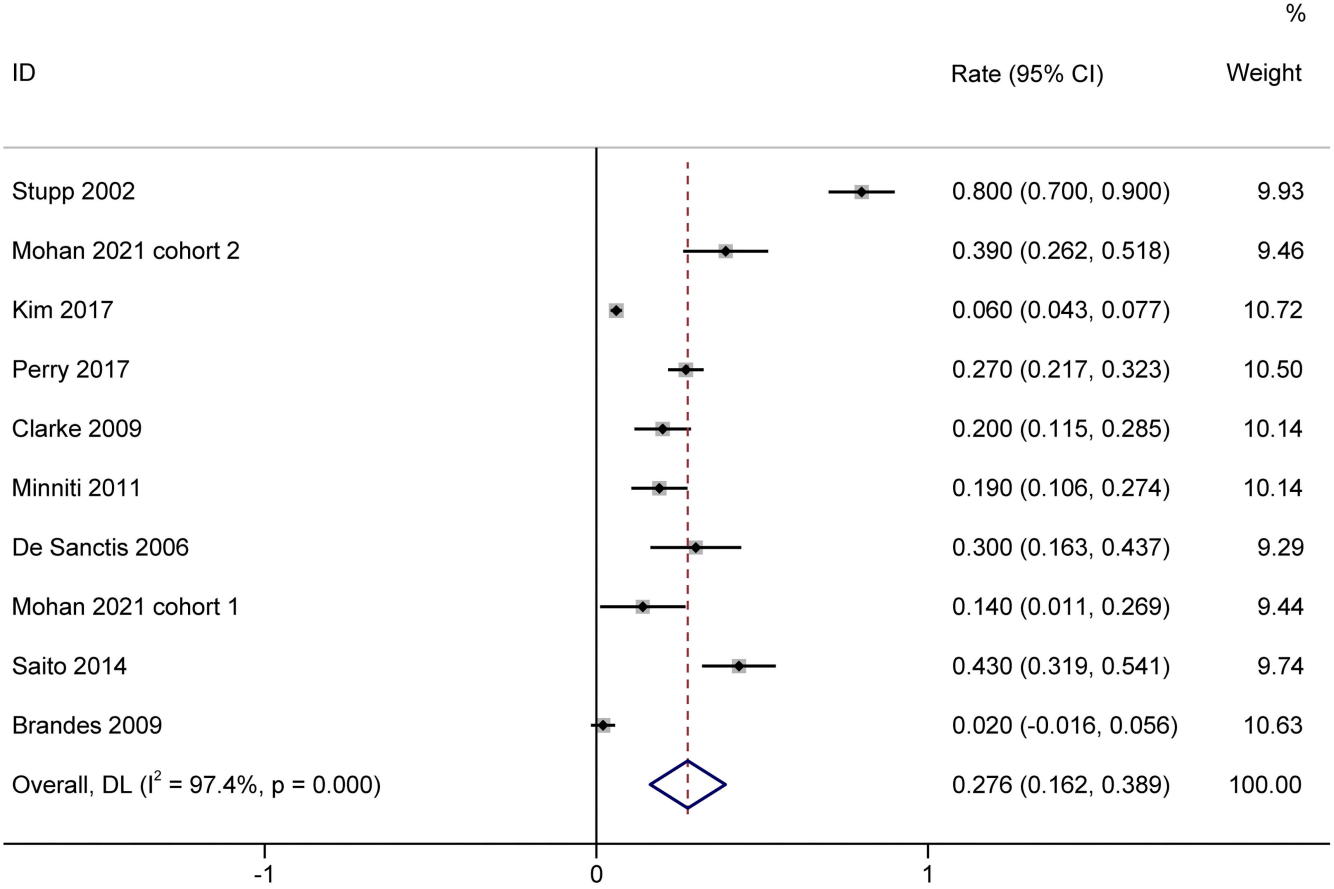
Forest plots of the incidence rate of severe lymphopenia and 95% CIs in GBM glioma patients. HR, hazard ratio; CI, confidence Interval; GBM, Glioblastoma. Weights are from random-effects model.

A total of 1,944 patients with newly diagnosed glioma who were treated with concurrent RT/TMZ and adjuvant monthly TMZ were included in this analysis. The incidence of high-grade (i.e., III/IV) lymphopenia in these studies was 31.6% (95%CI: 22.3−40.8%) (Figure 2). Moreover, as several studies including multiple types of glioma (e.g., astrocytoma, oligodendroglioma, and GBM), pooled results based on the pathology of glioma revealed that the incidence in astrocytoma and astrocytoma oligodendroglioma patients was 20.2% (95% CI:5.9–34.4%) (Figure 3), while that in GBM patients was 27.6% (95% CI:16.2–38.9%) (Figure 4).

### Impact of Lymphopenia on OS

**Figure 5:**
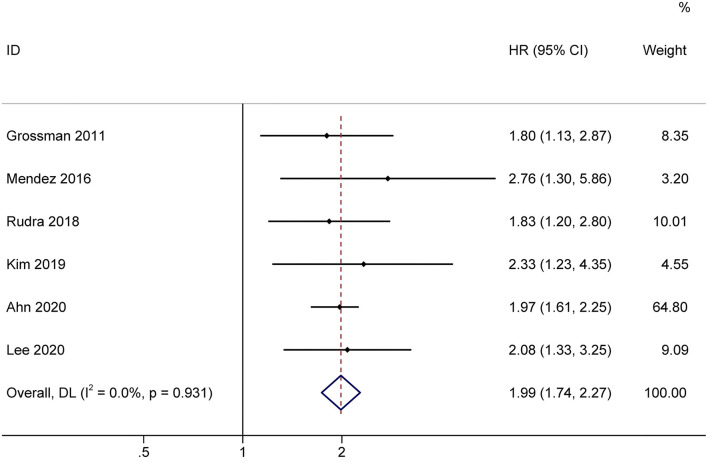
Forest plots of the prognostic impact of treatment-related lymphopenia on overall survival in glioma patients. HR, hazard ratio; CI, confidence Interval. Weights are from random-effects model.

The effect of treatment-related lymphopenia on OS was evaluated in 819 patients with newly diagnosed gliomas. Among them, one study of patients with various malignant gliomas was prospective, while the others that included GBM were retrospective. Most studies included patients with a median age of 54.2–59 years, while only Mendez et al. (13) included elderly GBM patients (age ≥ 65 years). The pooled results showed that patients with treatment-related lymphopenia had decreased survival (HR, 1.99; 95% CI, 1.74–2.27; *P*<*0.001*) compared to patients without lymphopenia (Figure 5). Similarly, lymphopenia was associated with inferior survival in patients with GBM after the exclusion of studies of patients with various malignant gliomas (HR, 2.00; 95% CI, 1.74–2.31; *P*< *0.001*). We further conducted subgroup analyses of patients with GBM. Subgroup analyses by sex (female <50 vs. female ≥ 50%) showed that lymphopenia led to poor OS for patients in both subgroups (female <50%: HR = 2.01 95% CI = 1.53–2.66, *P*< *0.001*; female ≥ 50%: HR = 2.00, 95% CI = 1.74–2.31, *P*< *0.001*). The results of subgroups based on steroid use (<50 vs. ≥50%) demonstrated that lymphopenia led to poor OS in both subgroups (steroid use <50%: HR = 1.98, 95% CI = 1.70–2.32, *P*< *0.001*; steroid use ≥ 50%: HR = 2.03, 95% CI = 1.75–2.35, *P*< *0.001*).

### Risk Factors for Lymphopenia

Five studies evaluated dosimetric parameters and other risk factors for lymphopenia (Table 3). Three studies reported that brain volume (receiving 25 or 20 Gy) was associated with an increased risk of lymphopenia. Moreover, female sex (four studies), older age (two studies) and lower baseline lymphocyte count (three studies) were also associated with increased risk of lymphopenia. Notably, several studies reported that baseline steroid use was not significantly associated with lymphopenia development, while a dexamethasone dose > 2 mg during RT was associated with an increased risk of lymphopenia.

**Table 3:** Risk Factors for developing Lymphopenia on Multivariate logistic regression analysis.

**ID**	**Dosimetric factors**	**Others**
Ishikawa et al. (21)	NA	Lower pre lymph (>1.2 ×10^3^/ ml), OR = 13.2 (1.25–143)
Huang et al. (33)	Higher brain volume receiving 25 Gy (OR: 1.03; 95% CI: 1.003–1.05). Brain V25 Gy <56% appeared to be the optimal threshold (OR: 2.36; 95% CI: 1.11–5.01)	Female sex [odds ratio (OR): 5.30; 95% confidence interval (CI): 2.46–11.41], older age (OR: 1.05; 95% CI: 1.02–1.09), lower baseline TLC (OR: 0.92; 95% CI: 0.87–0.98), and higher brain volume receiving 25 Gy (V25Gy) (OR: 1.03; 95% CI: 1.003–1.05)
Rudra et al. (31)	Higher brain V25 Gy (OR 1.048, 95% CI 1.022–1.074)	Older age (OR 1.091, 95% CI 1.047–1.137), and female sex (OR 2.901, 95% CI 1. 391–6.047).
Lee et al. (32)	NA	Female sex (male vs. female, HR: 0.31, *p* = 0.003) and dexamethasone dose > 2 mg per day (HR: 2.85, *p* = 0.032) were independently associated with for acute lymphopenia (TLC <1,000 cells/μL) 4 weeks after completion of CCRT and, respectively), while use of dexamethasone regardless of dose was not associated with lymphopenia (HR 1.45, *p* = 0.238).
Mohan et al. (30)	Whole-brain V20 (OR 1.07, 95% CI: 1.03–1.13, *P* = 0.002)	Being female (OR 6.2, 95% CI: 1.95–22.4, *P* = 0.003), baseline ALC (OR 0.18, 95% CI: 0.05–0.51, *P* = 0.003).

*NA, not available; OR, odds ratio*.

## Discussion

This meta-analysis pooled 1,944 glioma patients treated with concurrent RT/TMZ and adjuvant monthly TMZ to calculate the incidence of high-grade lymphopenia. Moreover, a total of 819 malignant glioma patients from six studies were pooled to examine the impact of treatment-induced lymphopenia on OS. We found that patients with post-treatment lymphopenia had poor OS compared to those with normal lymphocyte counts.

Lymphocytes, an essential component of the tumor microenvironment, have attracted interest in the era of immunotherapy. A reduced lymphocyte count is reportedly associated with inferior response rates, an increased risk of recurrence, and worse OS across various cancers, before, during or after treatment (6). Our study further confirmed that a decreased total lymphocyte count is associated with poor clinical outcomes in case of glioma, although the underlying mechanism has not been fully elucidated. One potential explanation for this finding is that the decline in lymphocytes after cytotoxic antineoplastic therapy may enhance the ability of surviving tumors to evade the immune system. Lymphocytes are considered essential for the suppression of tumor development within the immune microenvironment and as regulators of the immune surveillance process, thereby leading to poor prognosis in high-grade gliomas (2, 14).

RT, TMZ, and steroids are known to be lymphotoxic treatments in the routine management of patients with glioma. RT has long been considered an essential contributor to treatment-related lymphopenia in the management of various patients. RT-induced lymphopenia was first described in a large study that estimated long-term lymphocytes in women with breast cancer in 1971 (34), and it has since been observed in various cancers, such as glioma, pancreatic, lung, head and neck, esophageal, cervical, and bladder cancers (11, 35–37). Various circulating lymphocyte subpopulations that are indispensable parts of the immune response to cancer cells are influenced by RT, although the CD4 count was initially noted in a prospective study. Campian et al. (38) demonstrated that lymphocyte subpopulations, including immune cells of innate and adaptive immunity, were reduced in GBM patients after RT. Notably, regulatory T cells), a lymphocyte subpopulation that suppresses and controls the immune response, increased in patients with medulloblastoma during chemoradiotherapy, but decreased afterward (39). A possible mechanism behind lymphopenia in the brain is that a 6-week course of RT delivers a lymphotoxic dose to 99% of circulating blood cells due to limited bone marrow or lymphoid tissue exposure during RT (8). Hypofractionated radiation have been proven to be lymphoprotective because they have less of an effect on circulating lymphocytes (40). Furthermore, a higher brain volume receiving either 25 or 20 Gy was associated with a higher risk of lymphopenia. Huang et al. (33) first identified the optimal cutoff value for brain V25 Gy as 56% based on an outcome-oriented cut point determination method. Rudra et al. (31) further confirmed brain V25 Gy as the most significant dosimetric predictor of lymphopenia in GBM. Mohan et al. (30) reported brain V20 Gy was the most significant dosimetric factor associated with lymphopenia and that proton therapy yielded a lower Brain V20 Gy than photon therapy. The variation between BrainV25Gy vs. Brain V20 Gy might be partly explained by the fact that 33% percent of the patients received proton therapy. A recent study reported that GBM patients treated with proton therapy had reduced irradiated brain volumes and thus severe lymphopenia compared to patients treated with photon therapy (30). Hence, it is plausible to modify RT treatment strategies to alleviate lymphopenia.

Interestingly, Mendez et al. (13) compared total lymphocyte counts over time in older patients with GBM who received RT doses > 45 Gy vs. RT doses ≤ 45 Gy. The results showed that patients developed similar lymphopenia regardless of the RT dose they received. Lymphocyte counts reduced significantly in both short course (≤ 45 Gy) and longer course (>45 Gy) RT groups. Baseline median lymphocyte counts were 800 cells/mm^3^ which decreased to 600 cells/mm3 in patients who received RT doses ≤ 45 Gy, while these lymphocyte counts were 1,200 cells/mm^3^ which fell to 750 cells/mm3 in patients who received RT doses >45 Gy. The lower level of baseline lymphocytes in patients who received short course of RT may be a poor prognostic factor responsible for the shorter survival in these patients. These results are likely due to the small sample size, and an older patient population may have a higher risk of lymphopenia even with a short course of RT.

TMZ, which is associated with myelosuppressive toxicity, also causes lymphopenia. Previous studies reported that lymphopenia occurred in melanoma patients who were administered TMZ alone with a similar dose regimen to GBM patients who received RT/TMZ. A total of 29 (33%) patients developed grade III/IV lymphopenia, while 17 who discontinued TMZ after developing lymphopenia recovered normal lymphocyte counts in the range of 9 to 245+ days (41). The concurrent use of TMZ with RT increased the incidence of lymphopenia compared with RT alone. Perry et al. (28) reported grade III/IV lymphopenia in 10.3% of GBM patients given a shortened 3-week course of RT alone vs. 27.3% with the combination. Notably, Lin et al. (29) reported that concurrent TMZ during RT was the dominant factor resulting in lymphopenia within 3 months in younger patients with less aggressive grade II–III glioma. No cases of acute grade 3 lymphopenia after RT were observed among patients who did not received concurrent TMZ, and lymphopenia gradually mitigated over time in patients who received concurrent TMZ but no additional adjuvant TMZ. The role of concurrent or adjuvant TMZ in the development of lymphopenia in patients with GBM are requires further investigation.

Glucocorticoids are another commonly used lymphotoxic drug to mitigate edema in GBM patients before and after neurosurgery. Although glucocorticoids are considered immunosuppressive, their effects may be modest and dose-dependent. Several studies previously reported that baseline corticosteroid use was not significantly associated with an increased risk of lymphopenia after considering other confounding factors (e.g., tumor burden and radiation dose) (29, 31, 33). However, recent studies reported that cumulative dexamethasone dose is an independent risk factor for the development of lymphopenia during chemoradiation (32). In addition, the association between steroid use and clinical outcomes is controversial and may be affected by lymphopenia. In a recent meta-analysis evaluating the effects of steroids on outcomes in GBM patients treated with RT and/or TMZ, steroids significantly reduced OS and PFS (42). However, steroid use was not significantly associated with survival in our included studies (12, 13, 15, 16, 31, 32) when lymphopenia was included in multivariate models, suggesting that lymphopenia may be a significant adverse prognostic factor for OS.

In addition to the above factors, several baseline factors associated with an increased risk of lymphopenia were identified in various studies. As expected, patients who developed grade III–IV lymphopenia tended to have lower total lymphocyte counts before chemoradiotherapy. Lower lymphocyte counts or even lymphopenia might be a predisposing factor for tumor development and have been observed at the time of GBM diagnosis. Kim et al. (15) showed decreased levels of lymphocytes without steroid use at baseline, implying that lymphopenia is a frequent event in the natural progression of GBM. In addition to lower baseline lymphocyte counts, three studies reported that female sex is strongly associated with an increased risk of lymphopenia. In part, women have greater exposure of circulating lymphocytes to radiation as a higher rate of regional cerebral perfusion than man. Moreover, this sex-based phenomenon plays an important role in response to chemotherapy likely due to discrepancies in pharmacokinetics and pharmacodynamics between the sexes. Older age may reduce lymphocyte count due to telomere shortening and result in poorer physical condition. Currently, only one study included elderly patients with GBM (age ≥ 65) (13). This study showed that lymphopenia timing, severity, and duration were similar to those younger patients.

Our study has several limitations. First, most of the eligible studies were retrospective; thus, it is difficult to determine causation of lymphocytopenia among the volume of radiation, fractionations involved, TMZ, steroid use, and other possible confounding factors. Second, the heterogeneity of glial histological subtypes, the definition of lymphopenia and time of evaluation varied across different studies might have affected the stability and reliability of our analytical results. Third, most studies were unable to investigate the association between molecular markers and lymphopenia in the currently molecularly driven management paradigms, and only one study (13) reported there no association between MGMT and lymphopenia. Future prospective studies are required to incorporate molecular analysis to confirm our findings. Given the heterogeneity among studies, it should be considered hypothesis-generating. This review evaluates the impact of treatment-induced lymphopenia in HGG, and establishes the need for strategies to mitigate lymphopenia in these patients.

In conclusion, our study showed that post-treatment lymphopenia was associated with poor prognosis in high-grade glioma patients treated with RT/TMZ. Female sex, older age, brain receiving radiation dose of 20 or 25 Gy, and pretreatment lymphocyte levels are surrogate markers that predict lymphopenia, and minimizing them can reduce the lymphopenia and potentially improve the treatment outcomes of glioma patients, particularly in the current era of immunotherapy.

## Data Availability Statement

The raw data supporting the conclusions of this article will be made available by the authors, without undue reservation.

## Author Contributions

WL and EF: conception or design. HC, ShaC, and ZL: collection and/or assembly of data. YZ and ShiC: data analysis, interpretation, and manuscript writing. WL and EF: revision. YZ, ShiC, HC, ShaC, ZL, WL, and EF: final approval of manuscript.

## Funding

This study was supported by Capital's Funds for Health Improvement and Research (Grant Number 2020-2475 2175) and Beijing Talents Project.

## Conflict of Interest

The authors declare that the research was conducted in the absence of any commercial or financial relationships that could be construed as a potential conflict of interest.

## Publisher's Note

All claims expressed in this article are solely those of the authors and do not necessarily represent those of their affiliated organizations, or those of the publisher, the editors and the reviewers. Any product that may be evaluated in this article, or claim that may be made by its manufacturer, is not guaranteed or endorsed by the publisher.
