# Supplementary material for: Prognostic Value and Risk Factors of Treatment-Related Lymphopenia in Malignant Glioma Patients Treated With Chemoradiotherapy: A Systematic Review and Meta-Analysis
Source: Front Neurol. 2022 Jan 4;12:726561. doi: 10.3389/fneur.2021.726561 (PMC8764122; doi:10.3389/fneur.2021.726561)
Supplement: Supplementary file 1 [file Table_1.DOCX]

| **Table S1 Search strategy** | | |
| --- | --- | --- |
| Glioma | **#1** | ("Glioma"[Mesh]) OR (glioma*[Title/Abstract] OR astrocytoma*[Title/Abstract] OR medulloblastoma*[Title/Abstract] OR ependymoma*[Title/Abstract] OR craniopharyngioma*[Title/Abstract] OR oligodendroglioma*[Title/Abstract] OR glioblastoma*[Title/Abstract] OR GBM*[Title/Abstract]) |
| Radiotherapy | **#2** | ("Radiotherapy"[Mesh] OR radiotherap*[Title/Abstract] OR radiat*[Title/Abstract] OR irradiat*[Title/Abstract] OR RT[Title/Abstract])) OR ((radiochemo*[Title/Abstract] OR chemoradio*[Title/Abstract] OR chemotherap*[Title/Abstract] OR "Chemoradiotherapy"[Mesh] |
| Temozolomide | **#3** | (Temozolomide[Title/Abstract] OR TMZ[Title/Abstract]) |
| Lymphopenia | **#4** | ("Lymphopenia"[Mesh]) OR lymphocyte[Title/Abstract]) OR (Lymphopenia[Title/Abstract]) |
| Search strategy |  | #1 and (#2 or #3) and #4 |
